# Preparing for COVID-19: rapid redeployment workshops for Senior Doctors

**DOI:** 10.15694/mep.2020.000209.1

**Published:** 2020-09-28

**Authors:** Nicholas Wroe, Amy Tulip, Jake Wright, Rebecca Filewood, Emma Jones, Eleanor Owen, Kelly Murphy, Fiona Coia, Terasa Broom, Reshad Khodabocus

**Affiliations:** 1 Mid Yorkshire NHS Trust

**Keywords:** COVID-19, Simulation, Postgraduate education, Resilience, Adaptability, Lean working, Clinical Fellow, Junior doctors, Skill fade

## Abstract

This article was migrated. The article was marked as recommended.

Background

Restructuring secondary care provision for COVID-19 raised the possibility of redeployment of senior physicians. Increasing specialization meant that redeployment of non-acute or non-medical consultants to support the medical take was a source of anxiety.

Objective

We delivered focused refresher training for senior doctors. This study hoped to determine usefulness, feasibility and acceptability of delivering training in this new fashion.

Methods

Candidates undertook a half-day course of high-fidelity simulation, resuscitation, recognizing COVID-19, oxygen therapy, basic procedures, IT training, and PPE. The sessions were delivered by clinicians from across the medical and nursing hierarchy, with social distancing maintained throughout. All candidates were given an anonymous post-course evaluation.

Results

We received 307/360 evaluation forms. 98.7% of candidates agreed (31.1%) or strongly agreed (67.5%) that the course was beneficial. Candidates commented that they felt more confident, and less anxious, about redeployment to manage COVID-19 patients.

Discussion

The employment of Clinical Fellows in Medical Education without ties to service provision allowed them to focus on high volume, high quality training. The resultant redundancy in staffing proved useful in covering faculty sickness but also ensuring smooth running of the course. Freeing up the education team allowed simultaneous planning and adaptation of the sessions upskill 4 ^th^ and 5 ^th^ year medical students. Our simple course model with nimble staffing solutions could be reused in any future major incident.

Conclusions

Our experience demonstrates clear benefit in a cohort of juniors with educational interest. Lean working provides adaptability and resilience when training must be delivered rapidly.

## Introduction

The nationwide changes to hospital infrastructure to expand critical care capacity in anticipation of COVID-19 exposed the limitations of the current silo-working within medical specialisation. Experienced clinicians from non-acute specialties found themselves essentially furloughed due to reductions in elective work. In tandem with this, the prospect of redeployment to support the acute medical take as proposed by the GMC ( GMC, 2020) was a source of further stress and anxiety, reflected by the tone of the BMJ’s question and answer article ( Rimmer, 2020). While intensive care received large amounts of publicity, the majority of ‘front line’ work involved caring for COVID-19 patients at ward level, in great numbers. This was to be provided by redeployed, or even retired, doctors from all specialties. We identified that skill fade would be significant after years away from the acute floor, and that this was likely contributing to staff anxiety. To bridge this gap, we conducted Trust-wide redeployment training catered to this group.

We aimed to deliver focused refresher training on acute medicine and up-to-date knowledge and practices surrounding COVID-19, for senior doctors in non-acute medical and surgical specialties. This study hoped to determine usefulness, feasibility and acceptability of delivering training in this new fashion.

## Methods

This training was delivered as a half-day mandatory course, on multiple dates between 30 ^th^ March 2020 and 17 ^th^ April 2020. Notably, the course began only one week after the “Lockdown” declared by the UK government on 23 ^rd^ March 2020.

Candidates rotated around six half-hour workshops as shown in Table 1, including a high fidelity simulation of a deteriorating ward patient with Acute Respiratory Distress Syndrome, adaptations to resuscitation algorithms, up-to-date clinical knowledge surrounding COVID-19, oxygen therapy and arterial blood gas interpretation, clinical skills refresher on venepuncture, cannulation and ABG sampling, IT training surrounding ‘junior doctor’ tasks such as discharge letters and requesting investigations, and training in the safe donning and doffing of PPE. These were delivered in a socially-distanced fashioned, using an escorted closed-loop rotation system in groups of six candidates.

**Table 1.** Course Structure

Session	Format	Staffing
Recognition and Oxygen Therapy	Lecture	x2 Clinical Fellows in Medical EducationX1 Consultant Emergency Medicine
Simulation	High-fidelity simulation	Simulation Technician x1(Clinical Fellows as backup) Clinical Teachers (and ED Consultant) Volunteer clinicians (ICU registrars, consultant elderly medicine
Resuscitation	Resus Training	Trust resuscitation team x2
IT and Discharge Letters	IT Workshop	x2 Clinical Fellows in Medical Education
PPE	Practical Training	Matron for Medical Education
Clinical Skills	Venepuncture manikins	Clinical Teachers x3 Clinical Fellows as backup

The sessions were delivered by the Undergraduate Medical Education team, Trust Resuscitation Department and volunteer clinicians from Anaesthesia/ICU. The Undergraduate Medical Education team itself is diverse, including consultants in Emergency Medicine, Clinical Teachers in full-time educational roles from both medical and nursing backgrounds, and seven Clinical Fellows, junior doctors at either post-FY2 or ST3 level whose time is divided between clinical and educational work. We planned redundancy into the workshop staffing, to allow for faculty sickness/self-isolation, which was required on one occasion. A closed rotation system was used, and social distancing maintained in all workshops, with alcohol gel available in all rooms. Candidates were grouped by Division (“Medicine”, “Surgery”) to standardize prior knowledge where possible. All candidates were given an anonymous post-course evaluation (available in Supplementary File 1), with the option to declare their clinical specialty, the results of which form this study.

Ethical approval was sought from the Mid Yorkshire Research and Innovation Team, where the study was deemed exempt from formal panel review. Written permission for inclusion of candidate data was obtained.

## Results/Analysis

**Figure 1.**
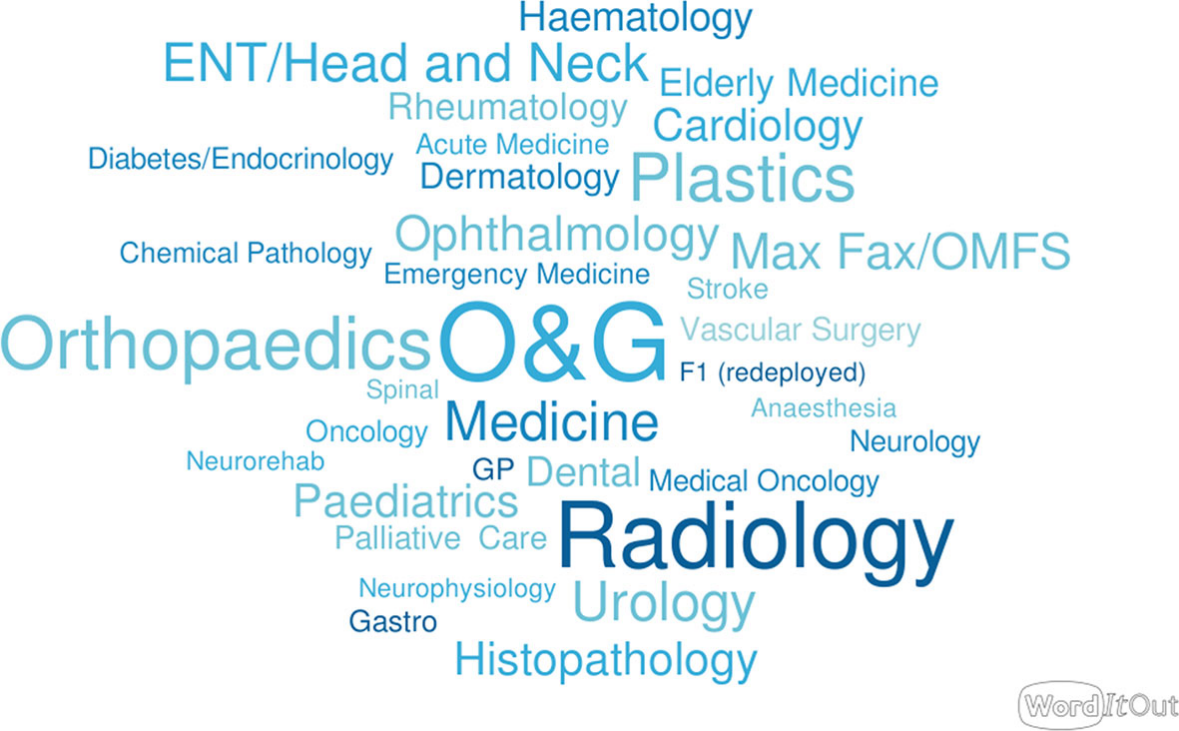
Proportion of specialties trained (Original data, word cloud by WordItOut, 2020)

**Figure 2.**
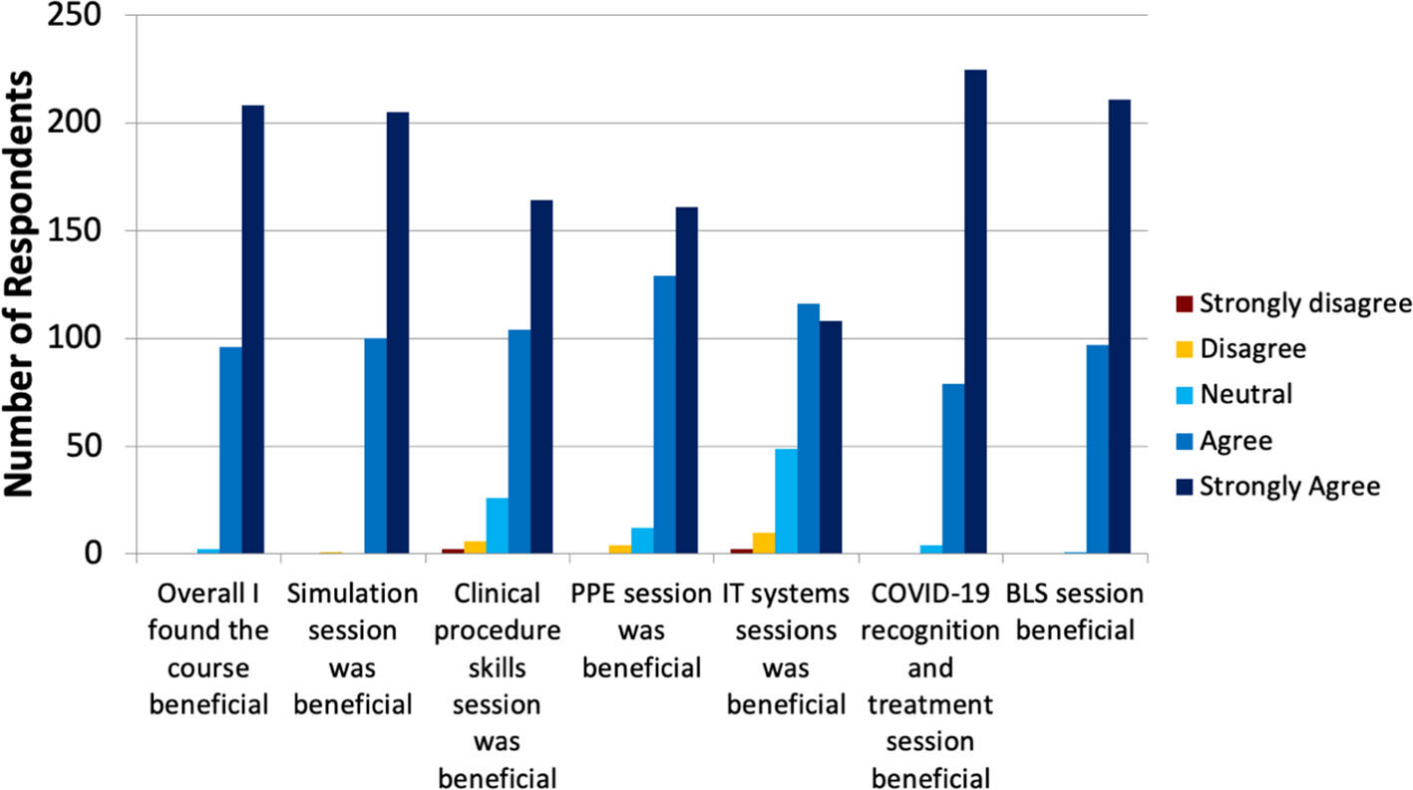
Course feedback broken down by session

We were able to train 360 clinicians across a range of specialties as broken down in Figure 1 ( WordItOut, 2020). Of these 360, 307 completed evaluation forms. Feedback was overwhelmingly positive, with 98.7% of candidates either agreeing (31.1%) or strongly agreeing (67.5%) that the course was beneficial. A full breakdown of feedback by session can be found in Figure 2. Positive qualitative feedback praised the organisation and structure of the course including chaperoning between stations, knowledge of the faculty, and the ability of the course to cater “at the right level” for a range of specialties, making it a “high yield afternoon”. Candidates also commented that they felt more confident, and less anxious, about the prospect of redeployment to manage COVID-19 patients. Negative comments were largely logistical, including the use of a video rather than practice in donning and doffing PPE (it is important to note the context of a national and international shortage of PPE at the time of the course) and the challenges of maintaining social distancing within the education centre’s geography. One candidate suggested the presence of senior management may have added authenticity to the training.

## Discussion

The intended outcomes of the course were threefold: to provide skills-based training, to provide standardized, validated information on topics such as PPE, where rumours and misinformation had proved problematic within the hospital, and to provide a forum whereby clinicians could express their concerns about redeployment and ask questions, with the aim of alleviating their anxiety. These goals were interdependent: it has been shown that conflicting information generates anxiety ( Hameen-Anttila *et al.*, 2014) and in the context of COVID-19 there were a number of areas where this was the case, most notably in the disagreements between Public Health England and the UK Resuscitation Council ( Resus Council UK, 2020), with subsequent implications for PPE requirements. We were able to assuage some of this anxiety by providing a one-stop-shop source of locally applicable, Trust-endorsed message which was consistent with both hospital and national guidance. Keeping clinicians broadly within their specialty groups made sense both pedagogically, making it easier to pitch sessions at a level appropriate to each group (a group of surgeons who routinely manage emergency laparotomies has a number of transferable skills, for example, compared to a non-acute specialty such as pathology), but also in providing camaraderie and creating a learning environment where candidates felt that their worries were shared and understood, again aiming to reduce anxiety around redeployment.

In our hospital as in many others, the number of junior doctors vastly exceeds that of consultants, and as a result it makes sense that juniors are empowered to drive change. Our Medical Education department employs seven Clinical Fellows in Medical Education, with no ties to clinical service provision. To our knowledge, this arrangement is not reproduced elsewhere in the UK, and was maintained despite pressure from hospital management. While the Clinical Fellows conduct locum work within the hospital in a variety of specialties, they were able to increase their educational time and mobilize to solve this training gap, which we strongly suspect is responsible for the speed, volume and quality of the training provided. These juniors were uniquely placed, with educational training, recent acute medical experience, and appropriate senior support, to provide high-quality training in a focused way. The nature of the sessions, whereby power had been removed from senior physicians due to enter an unfamiliar workplace, served to flatten power gradients and allowed junior members of the team to teach about the areas they knew best, such as discharge letters and the use of hospital systems.

Being a ‘Senior Decision Maker’ is dependent on logistical and organizational familiarity. Our training aimed to bridge these issues, allowing senior doctors to function not just in fulfilling junior-level functions in their redeployed wards, but as senior decision makers. This makes much better use of their advanced postgraduate training and experience in leadership and inter-specialty working.

The planned redundancy in staffing proved useful both in the event of faculty sickness, but also in freeing up staff to coordinate and ensure smooth running of the course. Freeing up members of the education team also allowed simultaneous planning and adaptation of the sessions. This proved time-critical as the format and content of the course was adapted over the subsequent two weeks to provide induction content to both 23 interim Foundation ‘FiY1’ doctors, registered early by the GMC to bolster the workforce, and a cohort of 44 fourth year medical students employed as ‘Physician Helpers’ to assist with basic procedures such as venepuncture/cannulation and administrative tasks. Our course design exemplifies the principles of lean working espoused by NHS Improvement ( Westwood, James-Moore and Cooke, 2007), whereby a simple course model with nimble staffing solutions could be reused in any future major incident, where redeployment or specific knowledge needs to be disseminated rapidly. In a similar fashion, such training could easily be adapted across the MDT, for example to support the redeployment of nursing staff into unfamiliar departments. Application of lean principles has precedent within the wider context of higher education ( Doman, 2011) and has potential to improve postgraduate medical education more widely: for example, in managing the challenges of time pressures of delivering training to Foundation trainees in the context of increasing service demands.

As the pandemic wanes and we look to the future, silo working cannot continue. The acute simulation part of our training was especially well received, and we received a number of suggestions of annual refresher training, such as is provided for resuscitation. This experience has unveiled the extent of skill fade within senior NHS clinicians across a range of specialties and identified an opportunity for postgraduate medical educators in reducing it.

## Conclusion

Despite these workshops being developed at rapid speed with minimal formal lesson planning, candidates’ feedback was universally positive. These sessions were designed with versatility in mind and were quickly adapted to deliver further training to upskill 4 ^th^ and 5 ^th^ year MBChB students. We demonstrate a clear benefit in employing a cohort of junior doctors with an educational interest, without ties to a clinical department, and highlight the merits of lean working to provide adaptability and resilience when training must be delivered rapidly. There is a gap to be addressed in providing iterative refresher training on emergency care for all specialties.

## Take Home Messages

• Lean working strategies can build resilience into a teaching programme, proving useful in fast-moving situations such as major incidents.
• Liberating Clinical Fellows in Medical Education from mandatory clinical commitments can pay dividends when innovation in teaching is required.

## Notes On Contributors

**Nicholas Wroe** is Clinical Fellow in medical education and simulation at Mid Yorkshire NHS Trust, and is about to commence ACCS Anaesthesia training. ORCID ID: https://orcid.org/0000-0003-0145-1279

**Amy Tulip** is a Clinical Fellow in medical education and simulation at Mid Yorkshire NHS Trust, and is about to commence GP training

**Jake Wright** is a Clinical Fellow in medical education and simulation at Mid Yorkshire NHS Trust, and is about to commence Paediatrics training.

**Rebecca Filewood** is a Clinical Fellow in medical education and simulation at Mid Yorkshire NHS Trust, and is about to commence GP training.

**Emma Jones** is a Clinical Fellow in medical education and simulation at Mid Yorkshire NHS Trust. She is continuing her role next year as a Senior Clinical Fellow.

**Eleanor Owen** is a Clinical Fellow in medical education and simulation at Mid Yorkshire NHS Trust. She is continuing her role next year as a Senior Clinical Fellow.

**Kelly Murphy** is a Senior Clinical Fellow in medical education and simulation at Mid Yorkshire NHS Trust. She is continuing her role next year as Lead Clinical Fellow in conjunction with GP training.

**Fiona Coia** is a Senior Clinical Teacher Mid Yorkshire NHS Trust. A nurse by background, she is responsible for coordinating and delivering undergraduate teaching to medical students of all years.

**Terasa Broom** is Simulation Lead at Mid Yorkshire NHS Trust, working in the department for Medical Education. She is also a consultant in Emergency Medicine at Mid Yorkshire NHS Trust.

**Reshad Khodabocus** is the Deputy Director of Medical Education, Mid Yorkshire NHS Trust. He is also a consultant in Emergency Medicine at Mid Yorkshire NHS Trust.

## Declarations

The author has declared that there are no conflicts of interest.

## Ethics Statement

Ethical approval was sought from the Mid Yorkshire Research and Innovation Group (our Trust’s audit/research body). It was deemed to be a service evaluation and therefore exempt from formal Ethics Panel review. Written consent for publication of data was obtained from all course candidates.

## External Funding

This article has not had any External Funding
