# Supplementary material for: Preparing for COVID-19: rapid redeployment workshops for Senior Doctors
Source: MedEdPublish (2016). 2020 Sep 28;9:209. [Version 1] doi: 10.15694/mep.2020.000209.1 (PMC10699367; doi:10.15694/mep.2020.000209.1)
Supplement: Peer review history

# Reviewer report: Reviewer response for version 1

**DOI:** 10.21956/mep.20127.r31060

**Published:** 2020-10-05

**Authors:** Haipeng Xiao

**Affiliations:** 1 The First Affiliated Hospital, Sun Yat-sen University

This review has been migrated. The reviewer awarded 4 stars out of 5 The authors presented an effective response strategy for staff training against COVID-19 early in the outbreak in the form of rapid redeployment workshops. Their work is especially commendable in creatively designing an educational intervention to facilitate the mobilisation of all personnel including (and in particular) senior physicians from all specialties to support acute medical take. While agreeing with what Trudie Roberts had proposed, an exploration of the learning experiences of these senior physicians might further increase the scientific merits of this paper, I would also suggest that it would perhaps be better, in the sense of presenting a more nuanced picture, to have included the assessment results of these trainees immediately after exposure, and, if possible, relevant outcomes of patients being cared for after the training. Overall, I highly recommended this paper for all who are in the same trenches.

Reviewer Expertise:

NA

No decision status is available

# Reviewer report: Reviewer response for version 1

**DOI:** 10.21956/mep.20127.r31061

**Published:** 2020-09-28

**Authors:** Trudie Roberts

**Affiliations:** 1 Leeds Institute of Medical Education

This review has been migrated. The reviewer awarded 5 stars out of 5 I enjoyed reading this paper and although it reported a straightforward initiative it is of practical importance. As the authors state their staffing configuration allowed them to be agile in their response to supporting patient safety at a time of medical redeployment which was very stress for all staff. I like the idea of 'reverse peer teaching'. It would have been interesting to explore how the senior clinicians felt being trained by junior trainees. I suspect it was welcomed and increased a feeling that 'they were all in this together'

Reviewer Expertise:

NA

No decision status is available
